# Clinical Characteristics and Potential Pathogenesis of Cardiac Necrotizing Enterocolitis in Neonates with Congenital Heart Disease: A Narrative Review

**DOI:** 10.3390/jcm11143987

**Published:** 2022-07-09

**Authors:** Kathryn Y. Burge, Aarthi Gunasekaran, Marjorie M. Makoni, Arshid M. Mir, Harold M. Burkhart, Hala Chaaban

**Affiliations:** 1 Department of Pediatrics, Division of Neonatology, University of Oklahoma Health Sciences Center, Oklahoma City, OK 73104, USA; kathryn-burge@ouhsc.edu (K.Y.B.); aarthi_gunasekaran@mednax.com (A.G.); marjorie-makoni@ouhsc.edu (M.M.M.); 2 Department of Pediatrics, Division of Cardiology, University of Oklahoma Health Sciences Center, Oklahoma City, OK 73104, USA; arshid-mir@ouhsc.edu; 3 Department of Surgery, Division of Cardiovascular and Thoracic Surgery, University of Oklahoma Health Sciences Center, Oklahoma City, OK 73104, USA; harold-burkhart@ouhsc.edu

**Keywords:** necrotizing enterocolitis, congenital heart disease, neonatal, prematurity, cardiology, enteral feeding, hypoplastic left heart syndrome

## Abstract

Neonates with congenital heart disease (CHD) are at an increased risk of developing necrotizing enterocolitis (NEC), an acute inflammatory intestinal injury most commonly associated with preterm infants. The rarity of this complex disease, termed cardiac NEC, has resulted in a dearth of information on its pathophysiology. However, a higher incidence in term infants, effects on more distal regions of the intestine, and potentially a differential immune response may distinguish cardiac NEC as a distinct condition from the more common preterm, classical NEC. In this review, risk factors, differentiated from those of classical NEC, are discussed according to their potential contribution to the disease process, and a general pathogenesis is postulated for cardiac NEC. Additionally, biomarkers specific to cardiac NEC, clinical outcomes, and strategies for achieving enteral feeds are discussed. Working towards an understanding of the mechanisms underlying cardiac NEC may aid in future diagnosis of the condition and provide potential therapeutic targets.

## 1. Introduction

Necrotizing enterocolitis (NEC) is the most common gastrointestinal emergency in the neonatal intensive care unit (NICU), with mortality approaching 30% in infants born weighing less than 1500 g [1]. In preterm infants, an underdeveloped intestinal tract intersects with a hyperinflammatory and immature immune system to allow bacterial translocation of the intestinal mucosa, often leading to necrosis of the bowel and multisystem organ failure [2]. This classical, preterm NEC (classical NEC) often initiates with feeding intolerance and abdominal distention, but can progress rapidly to bowel perforation and sepsis. Congenital heart disease (CHD) is a known risk factor for NEC development in both the preterm [3] and term [4] populations, associated with nearly 20% of normal weight (>2500 g), term NEC cases [5]. However, despite its prominent association with term NEC, the contribution of CHD to intestinal pathology in infants is incompletely understood.

NEC in infants with CHD (cardiac NEC) was first reported in 1976, and is primarily associated with the ductal-dependent (DD) hypoplastic left heart syndrome (HLHS) [6]. Incidence of cardiac NEC, whether in term or preterm infants, is estimated at between 3 and 5% [7,8,9,10,11], with higher rates (between 6 and 9%) in infants with HLHS [7,12,13] and an inverse relationship with gestational age [14]. The true incidence of cardiac NEC is difficult to gauge, however, as most studies have involved only single centers [15]. Given its rarity, cardiac NEC has been difficult to study, and most reports are based upon retrospective analysis.

The presentation of cardiac NEC is known to differ from that of classical NEC in several respects. Infants with cardiac NEC are typically of higher birth weight and gestational age [15] than preterm infants with classical NEC. While some groups have argued that cardiac NEC more often involves the colon [16,17,18], the ileum, as with classical NEC, also appears to be a significant site of injury in cardiac NEC [19,20,21]. The postnatal age at which term CHD infants acquire NEC is often earlier than preterm infants with classical NEC [16], and preterm infants with DD cardiac NEC are often diagnosed at a later postnatal age than term infants with the same CHD diagnosis [22]. Cardiac NEC can occur either preoperatively or postoperatively, with mixed findings on the timing of NEC development in relation to cardiac surgery [23,24]. Interestingly, Lau et al. have documented that cardiac NEC, in association with non-DD CHD, typically occurs prior to cardiac surgery, while NEC associated with DD lesions frequently occurs postoperatively [7]. The authors speculate the more extensive surgery required of DD lesions may provide additional interruptions to intestinal blood supply. The use of prostaglandins (PGEs) in DD patients in order to boost or maintain peripheral perfusion [25] has been associated with the development of cardiac NEC. While the risk of developing NEC while receiving PGEs appears to be low [7,22], long duration of use [8,22] or high doses [10] of PGEs have been linked with cardiac NEC development. Compared with classical NEC infants, cardiac NEC infants often require less supportive critical care, such as vasopressors and respiratory support, but these supportive measures are likely associated, in part, with infant gestational age [23].

Outcomes in classical and cardiac NEC also differ. Pickard et al. compared secondary outcomes, including perforation of the bowel, stoma requirement, and additional operations, in cardiac and classical NEC, finding fewer secondary outcomes among cardiac NEC patients. However, this relationship dissipates if isolated-transitional patent ductus arteriosus (PDA), a diagnosis often not considered with cardiac NEC, is removed from the analysis [26]. While fewer cardiac NEC infants require surgical resection of the bowel, these infants may suffer higher all-cause mortality compared with classical NEC infants [9]. Rates of mortality due specifically to cardiac NEC are difficult to determine, as reports differ on the inclusion of suspected NEC, patients graded as Bell’s Stage I [15], but most clinicians consider the development of NEC to substantially increase the mortality risk in the setting of CHD [10,16,22,27,28], especially in the context of cyanotic disease [29].

## 2. Pathogenesis of Intestinal Injury in Congenital Heart Disease

**Figure 1:**
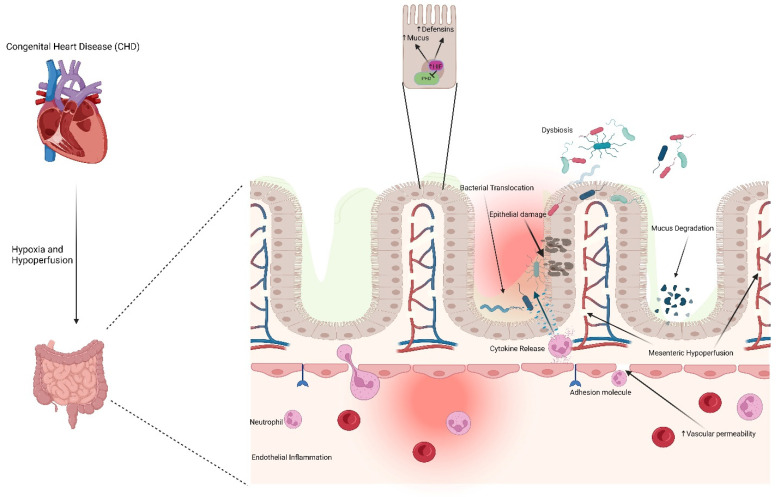
Speculated mechanisms contributing to cardiac NEC. Congenital heart disease induces mesenteric hypoperfusion and hypoxia in the gut, resulting in endothelial inflammation, vascular permeability, release of cytokines, epithelial damage, bacterial translocation, degradation of mucus, and dysbiosis. HIF: hypoxia inducible factor; PHD: prolyl hydroxylase domain; NEC: necrotizing enterocolitis. Created with Biorender.com (accessed on 6 July 2022).

Much like classical NEC [30], the pathophysiology of cardiac NEC is thought to be multifactorial (Figure 1), but with a significant emphasis on factors affecting perfusion of the gut [15]. Retrograde diastolic flow of the abdominal aorta is a common feature among many cardiac NEC patients. Carlo et al. demonstrated that 47% of cardiac NEC patients, compared with only 15% of the gestational age- and diagnosis-matched controls, suffered from impaired diastolic flow of the abdominal aorta [31]. This retrograde flow was present among a variety of CHD pathologies. Harrison et al. conducted ultrasound measurements of the superior mesenteric artery (SMA) resistive index, a proxy for perfusion, and diastolic flow reversal before and after modified Norwood palliation of HLHS [32], finding an impaired SMA perfusion and a high incidence of diastolic flow reversal. No changes in the SMA resistive index or incidence of retrograde diastolic flow were documented comparing pre- and postoperative readings. While no infants in this small study developed NEC, the lack of apparent improvement in postoperative splanchnic perfusion is a potential indication that underlying physiology predisposing these infants to the development of cardiac NEC was not resolved following initial CHD surgery. Further attempts to directly document the reduced mesenteric perfusion in developing cardiac NEC cases have focused on near-infrared spectroscopy (NIRS) readings, but variability due to small sample sizes, gut motility and feeding status, and age and size of the infants has limited the ability to extrapolate from these studies [33]. In addition to persistent hypoperfusion or retrograde diastolic flow, intermittent hypoperfusion, such as from shock or low cardiac output, has also been associated with cardiac NEC [10]. Another proposed component of splanchnic hypoperfusion in cardiac NEC infants is an inherent abnormality in the mesenteric vasculature, independent of the left ventricular dysfunction characteristic of HLHS. Miller et al. demonstrated lower abdominal aorta pulsatility indices in infants developing NEC compared with infants not developing NEC, both before and after the first-stage palliation of HLHS. The authors suggest that the subset of HLHS infants developing NEC likely suffered from systemic vasculature abnormalities prior to NEC development, a result of either primary pathology or unique alterations in blood flow secondary to HLHS [34].

In cardiac NEC infants, mesenteric hypoperfusion or intestinal ischemia may initiate as early as delivery in the transition from shared maternal circulation [35]. Cardiac NEC most frequently involves the colon and distal ileum due to the susceptibility to superior and inferior MA blood supply interruptions in these regions [36], often referred to as ‘watershed zones’ [35]. Mechanistically, mesenteric hypoperfusion initiates endothelial inflammation and increases vascular permeability and neutrophil, leukocyte, and platelet accumulation and activation due to endothelial production of adhesion molecules [37]. Cytokine release by endothelial cells exacerbates the inflammatory response, which spreads throughout the submucosa to the mucosa, increasing intestinal epithelial permeability [38]. Mesenteric ischemia also results in mucin degradation, leaving the gut mucosa vulnerable to autodigestion via pancreatic proteases. Importantly, this inflammatory process in cardiac NEC infants occurs on a background of comparatively higher systemic inflammatory cytokine and endotoxin levels, the latter a consequence of increased intestinal permeability [39,40], than in infants without CHD [41,42]. In cardiac NEC cases developing postoperatively, reperfusion injury spurred by corrective surgical restoration of blood flow, particularly in cases involving cardiopulmonary bypass (CPB) and hypothermia [40,43], contributes to alterations in blood flow patterns and furthers systemic inflammation [39], particularly through the generation of reactive oxygen species (ROS). In addition, red blood cell (RBC) transfusions have been independently associated with NEC development in CHD term infants postoperatively [4]. The postnatal timing of these surgical sources of inflammation or hypoperfusion may account for a portion of the age variability in cardiac NEC development.

Van der Heide et al. provided evidence for tissue hypoxia and ischemia in near-term CHD infants subsequently developing NEC. In the few days between birth and the development of NEC, these CHD infants were characterized by lower Apgar scores and increased respiratory support, with a trend toward lower pH and diastolic blood pressure, compared with age-matched CHD infants not developing NEC [35]. In addition, lower levels of platelets and C-reactive protein (CRP) in term NEC infants with CHD compared with preterm NEC infants without CHD provided further evidence of the ischemic nature of cardiac NEC in comparison with the more acute inflammatory classical NEC [18].

Characteristics of the neonatal intestinal vasculature relating to the developmental stage and regulatory capability likely predispose infants to cardiac NEC. Healthy newborn intestinal circulation, in order to allow for rapid growth [44], is characterized by a high rate of blood flow and oxygen delivery via low resting vascular resistance [45]. This low resistance is met through crosstalk between the intrinsic factors endothelin-1 (ET-1), largely a vasoconstrictor, and nitric oxide (NO), a vasodilator, with the balance favoring vasodilation in the resting newborn [45]. Neonatal vasodilation occurs, in part, due to enhanced MA response to, and increased concentration of, NO in the blood [46]. NO production is also driven by high blood flow [47], producing the vasodilation required to accommodate this high flow. A potential consequence of the newborn low vascular resistance is the inability to respond effectively to hypotension or arterial hypoxemia [36]. Neonatal animal models have demonstrated a maladaptive vasoconstriction in response to arterial hypoxemia, further reducing intestinal perfusion through ischemia [48]. This pathology occurs in the infant when the ET-1/NO balance, normally favoring NO-induced vasodilation, instead leans toward ET-1 and vasoconstriction. In severe cases of classical NEC, this signaling imbalance likely occurs as a secondary event via inflammation-associated endothelial cell dysfunction, altering the NO production. In cardiac NEC, however, microcirculatory alterations are likely a primary event [49], spurring hypoxia-induced ET-1 upregulation and resulting in secondary mucosal inflammation. Many of the microcirculatory changes in CHD infants are considered to be adaptive responses to chronic hypoxia. Increased viscosity of the blood, reduction in deformability of RBCs, increased blood vessel shear stress, changes in vasodilation, and circulatory remodeling are common in cyanotic CHD [49], and likely contribute to the pathogenesis of cardiac NEC. Thus, the neonatal intestine lacks the critical collateral networks and pressure-flow autoregulation of an adult, and when combined with a higher tissue basal metabolic rate, is far more susceptible to ischemia [50].

Additional factors thought to play a role in the pathogenesis of cardiac NEC are similar to those of classical NEC. Pathogenic bacteria likely to translocate the ischemically compromised intestinal epithelium in cardiac NEC originate from an altered microbiome, similar to that of classical NEC [51], but in the case of cardiac NEC, have likely proliferated due to intestinal hypoperfusion, venous congestion and bowel wall edema, and associated inflammation [52]. Infants with cardiac NEC harbor additional Firmicutes, but reduced Actinobacteria, Bacteroidetes, and Enterobacteriaceae, as well as reduced total bacterial counts, compared with healthy controls [53]. Delayed enteral feeds and antibiotic exposure can further exacerbate microbial dysbiosis [15]. Unfortunately, small clinical trials administering probiotics in infants undergoing CHD surgery have yet to demonstrate a reversal of this dysbiosis, nor significant benefits to the infant [53,54]. This enduring hypoperfusion-induced dysbiosis further weakens the intestinal barrier, allowing inflammatory molecules such as trimethylamine N-oxide (TMAO) to additionally activate the endothelium [52]. Low blood flow, in combination with the induced overgrowth of proinflammatory, facultative anaerobes spurred by this low oxygen environment, induce upregulation of hypoxia inducible factor-1 alpha (HIF-1α) and NF-κB (nuclear transcription factor-κB), the latter influenced by either a reduction in butyrate-producing organisms [52] or crosstalk with HIF-1α [55]. While both transcription factors upregulate genes with a protective role in intestinal barrier function, hypoxia-induced inflammation often results in increased intestinal permeability and cytokine production, resulting in excessive intestinal inflammation [56]. Increased HIF-1α results in a heightened expression of the downstream target, VEGF (vascular endothelial growth factor) [36], and erythropoietin (EPO) [57]. Increased VEGF expression may account for the high vascular density in some cyanotic CHD infants [49], while elevated RBCs may induce a reoxygenation injury in the anemic gut [58]. However, supraphysiologic oxygen supplementation following birth or surgical procedures in CHD infants may reverse HIF-1α elevations induced by hypoperfusion and dysbiosis, negating the adaptive response to hypoxia [56]. Thus, stabilizing HIF expression, as through pharmacological inhibition of the HIF-1α inhibitors, prolyl hydroxylase domain (PHD) proteins, has been suggested to be protective in neonatal populations susceptible to NEC development [57].

## 3. Risk Factors Contributing to Cardiac NEC Pathogenesis

Prematurity, low birth weight (LBW, <2500 g), high preoperative risk assessment scores, RBC transfusions (RBCTs), trisomy 21, and specific CHD pathologies may predispose infants to acquiring cardiac NEC (Table 1). Natarajan et al. demonstrated that even late-preterm infants, when compared with term, CHD diagnosis-matched controls, sustained a higher risk of NEC development [8]. In addition, specific cardiac lesions may pose a relatively insignificant risk of NEC development in term CHD infants, but the same diagnosis at a younger gestational age may result in a significantly worse outcome [59]. Similarly, low birth weight infants with CHD often appear at a higher risk of developing NEC compared with age-matched controls [22], and many LBW infants fare worse than larger infants of the same gestation age [13].

Elevated preoperative risk assessment scores have been associated with the development of cardiac NEC. The risk adjustment for congenital heart surgery (RACHS-1), an estimate of the complexity of the CHD diagnosis [60], is correlated with cardiac NEC risk when the values exceed 2 [7], likely due to time spent hypothermic or on CPB. Many CHD infants receive RBCTs during or following surgery. A retrospective association between RBCTs and classical NEC in premature infants has been recognized [2,58]. Baxi et al. observed an association between an increased RBCT rate in term CHD infants and the development of cardiac NEC [4], but, as with preterm infants, whether the administration of RBCTs or the underlying anemia is causally associated with cardiac NEC development is yet to be determined [61,62]. Infants with trisomy 21 suffer from immune [63] and gastrointestinal [64] anomalies, likely predisposing this population to the development of NEC [65,66]. In addition, these infants have extremely high rates of CHD, particularly atrioventricular septal defects (AVSDs) [27], estimated at over 50% incidence [67]. The consequences of AVSD, including shunted blood flow and cyanosis [27], almost certainly contribute to the pathogenesis of cardiac NEC in this population.

Cardiac NEC appears to be closely associated with several cardiac pathologies. Single ventricle defects, especially HLHS [7,10,13,68], account for the majority of CHDs associated with cardiac NEC. The mortality of single ventricle-associated cardiac NEC, approaching 25% with HLHS [13], is often a result of interplay with and among concomitant risk factors, such as prematurity and LBW. In a single-center study, Iannucci et al. found that 67% of term or near-term CHD infants developing NEC were diagnosed with single ventricle defects, while 55% of cardiac NEC patients had systemic outflow tract obstructions [24]. DD lesions, in general, have also been associated with the development of cardiac NEC, with an incidence estimated at 5% [7]. AVSD (among very LBW infants, VLBW, <1500 g) [27], truncus arteriosus, and aortopulmonary window (APW) [10] have also been independently associated with the development of NEC.

## 4. Abdominal Complications Relating to HLHS Stage I Palliation

**Figure 2:**
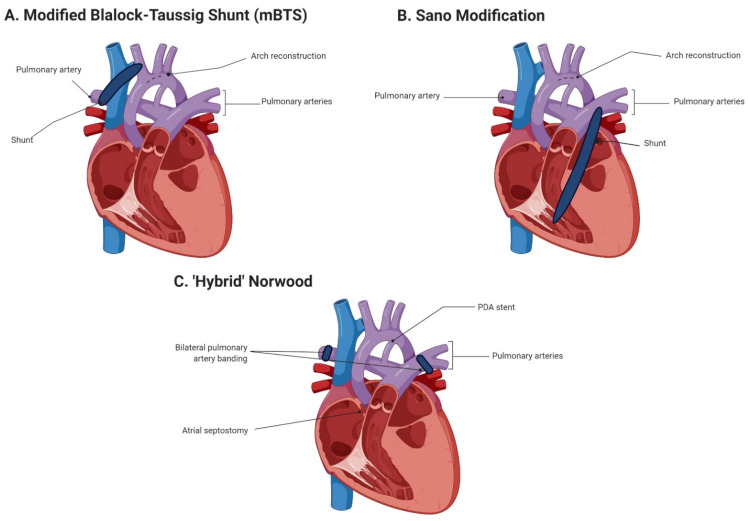
Current surgical options for Norwood Stage I palliation. PDA: patent ductus arteriosus. Created by Biorender.com (accessed on 3 June 2022).

The surgical procedure (Figure 2) selected to repair single ventricle defects in infants with HLHS is critical in that these infants comprise 67% of all cardiac NEC cases [10,24]. Infants with DD systemic circulation typically undergo Stage I Norwood palliation via reconstruction of the aortic arch, combined with either a subclavian or innominate artery to pulmonary artery shunt (modified Blalock–Taussig shunt (mBTS)) or a right ventricular to pulmonary artery shunt (Sano modification) [69]. The use of mBTS has been associated with decreased systemic perfusion due to retrograde diastolic flow through the SMA and aorta, similar to what is observed in premature infants with a hemodynamically significant PDA [15]. A high incidence of NEC following Stage I Norwood palliation with mBTS, potentially influenced by the shunt size-to-body weight ratio [70], has resulted in a surgical preference toward the Sano modification. The Sano modification is thought to reduce NEC risk via a reduction in retrograde diastolic flow [32,71,72,73], as well as potentially reduce the time to enteral feeds [71]. However, SMA perfusion following either Stage I method has been demonstrated to be impaired, compromising blood flow to the gut and escalating the risk of NEC development [74,75]. A third palliative approach is now available, utilizing bilateral banding of the pulmonary arteries and arterial duct stenting [76]. This less invasive ‘hybrid’ approach offers benefits in that CPB and hypothermic circulatory arrest are not required, reducing the risk of mesenteric ischemia, postoperative low cardiac output, and CPB-associated neurodevelopmental delays. Despite these advantages, the ‘hybrid’ approach does not appear to decrease the time to enteral feeding, carries a high rate of abdominal complications [77], does not improve postoperative hemodynamic stability or oxygen transport compared with traditional Norwood Stage I palliation [78], and thus far, does not appear to reduce risk of NEC [79]. However, an interventional transcatheter technique for bilateral pulmonary artery banding without the requirement for surgery has been recently described [80], providing a promising outlook.

## 5. Practices for Initiation and Maintenance of Enteral Feeds in Infants with CHD

Current clinical practices regarding the enteral feeding of CHD infants are based largely on cohort studies and practitioner preferences, with only a few multicenter, randomized, controlled trials available. The type, number, and initiation of feeds in infants at risk of classical NEC is a contentious subject [81], but the discussion is complicated further in the context of cardiac NEC risk [15]. In a healthy, fasted state, the gut requires less than 5% of total blood volume. Upon feeding, the requirement for blood to the gut increases to 30% to account for the metabolic activity associated with nutrient uptake and digestion [82], but the compromised vasculature and blood flow of an infant with CHD may not respond appropriately to this increased demand. As with classical NEC, human milk (HM) is likely protective, but additional factors inherent to hospitalization for CHD, including prolonged hospital length of stay (LOS) and additional caloric requirements, contribute to low rates of breastfeeding in these infants [83]. In addition, ambitious preterm growth targets often necessitate the use of HM fortifiers, increasing the solute concentration of the infant diet. Fortification of HM, based largely on animal studies [84,85], is a suggested risk factor for classical NEC [86], but the risks associated with increased dietary osmolality may be further extended in the setting of CHD-associated intestinal ischemia [87]. Cognata et al. demonstrated that an exclusively HM diet without fortification has been associated with a significant reduction in NEC risk preceding complex CHD repair [83]. However, due to the perception of increased risk for NEC development, and despite substantial evidence (e.g., [12,88]), even HM preoperative feeds are often controversial in cardiac NEC infants [83,89].

The perception of NEC risk upon enteral feeding is not unfounded, however, as abnormal SMA blood flow, both at baseline and postprandially, has been documented in single ventricle CHD infants, even after Stage I palliation [90]. Conflicting studies on SMA perfusion of the gut, particularly among Stage I palliation techniques, have further clouded the question of preoperative feeds. While intestinal hypoperfusion in CHD infants, especially DD infants using PGEs, is thought to limit the safety of larger feeds due to increased digestion-induced metabolic activity, a potential consequence of fasting infants during the preoperative period is villus atrophy [20,83]. Some centers have utilized trophic feeds in an attempt to minimize the NEC risk in CHD patients. Toms et al. demonstrated that 20–30 mL/kg/d trophic feeds, in comparison with nil per os (NPO), in term infants with HLHS awaiting Norwood palliation did not increase risk of NEC [91], but Cognata et al. noted that significantly larger feeding volumes (>100 mL/kg/d) increased the risk of NEC development in infants with complex CHD [83]. Scahill et al. also noted no increase in NEC incidence with greater than trophic (>20 mL/kg/d) compared with trophic (<20 mL/kg/d) preoperative feeds in infants requiring cardiac surgery, though the maximum feeding volume was not defined [12]. However, preoperative feeds, especially in HLHS infants, have largely been studied in a retrospective manner, without clear guidelines and with significant reliance on provider discretion.

**Figure 3:**
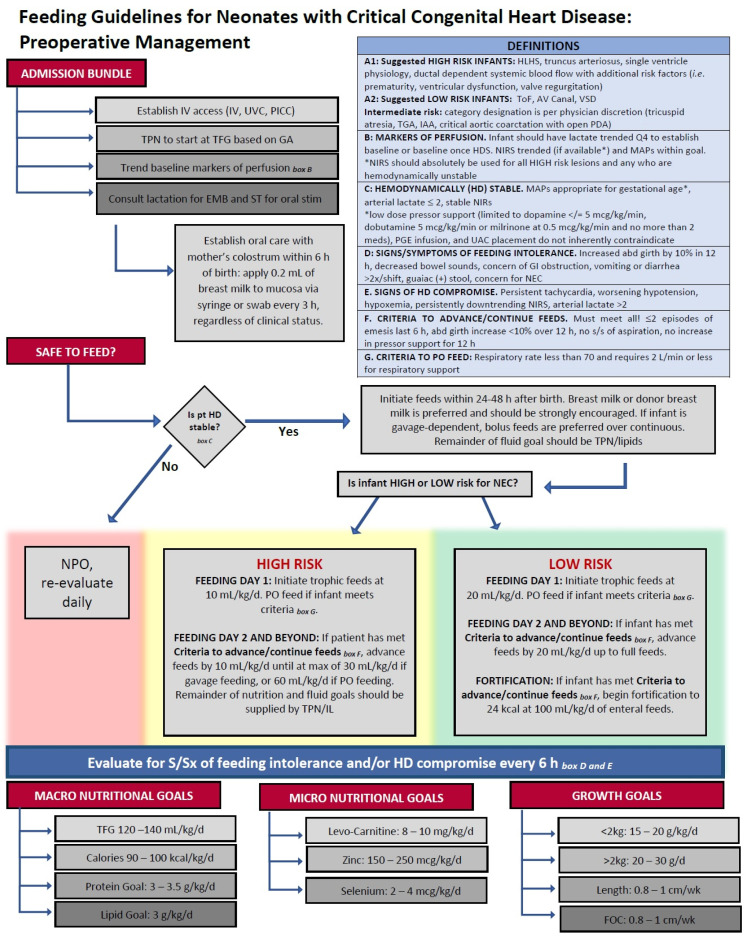
Representative preoperative standardized feeding protocol for neonates with congenital heart disease (CHD). IV: intravenous; UVC: umbilical venous catheter; PICC: peripherally inserted central catheter; TPN: total parenteral nutrition; IL: intralipid therapy; TFG: total fluid goal; GA: gestational age; EBM: expressed breast milk; ST: speech therapy; HLHS: hypoplastic left heart syndrome; ToF: tetralogy of Fallot; AV: atrioventricular; VSD: ventricular septal defect; TGA: transposition of the great arteries; IAA: interrupted aortic arch; PDA: patent ductus arteriosus; Q4: fourth quartile; HDS: hemodynamically stable; NIRS: near-infrared spectroscopy; MAPs: mean airway pressures; PGE: prostaglandin; UAC: umbilical arterial catheter; NEC: necrotizing enterocolitis; NPO: nil per os; PO: per os; S/Sx: signs and symptoms; FOC: fronto-occipital circumference.

In the United States and Europe, pre- and postoperative feeding practices for infants at risk of cardiac NEC have varied widely [92]. Most feeding practices in this population are extrapolated from clinical guidelines intended for critically ill neonates or those at risk of developing NEC, such as The American Society for Parenteral and Enteral Nutrition (ASPEN) guidelines [93]. In the CHD infant, cardiac diagnosis, PGE use and dose, utilization of umbilical venous or arterial catheters, and vasoactive drug administration are factors generally considered before feeds are initiated, while serum lactate levels, gastric residual volumes, and arterial blood gas oxygenation levels are commonly used benchmarks to assess readiness for feeds [94]. A 2017 survey conducted by The European Society of Pediatric and Neonatal Intensive Care indicated that only a third of pediatric intensive care units (PICUs) utilize preoperative or postoperative nutritional guidelines [94]. However, the use of standardized feeding protocols, rather than individual provider discretion, has been effective in improving patient outcomes [95,96,97,98]. In infants following HLHS Stage I palliation, del Castillo et al. found a significant decrease in NEC development when standardized protocols advancing HM or hydrolyzed formula feeds slowly over a 7 d period were followed, rather than individual provider discretion [75]. While those infants on standardized protocols required a longer period before full feeds, clear guidance on interruptions for feeding intolerance resulted in reduced hospital LOS. Furlong–Dillard et al. implemented a standardized feeding protocol in infants with complex CHD. When compared with previous variable practitioner preferences, the standardized protocol increased rates of preoperative feeding and reduced postoperative total parenteral nutrition (TPN), without increasing complications [97]. Based on available studies, our institution has developed standardized preoperative feeding protocols for neonates with critical CHD (Figure 3), focused largely on hemodynamic stability and the perceived risk of NEC development [97,99,100,101,102,103,104]. Globally, however, extreme variability in enteral feeding guidelines puts CHD neonates at risk of malnutrition, growth failure, and poor postoperative outcomes [105,106].

Given the high risk of NEC development following cardiac surgery, postoperative feeding strategies are heavily scrutinized in the CHD infant population, though clinical trials on this topic are lacking. Clearly, the resumption or initiation of feeds in these infants is not the sole source of NEC risk, as Iannucci et al. demonstrated that 27% of infants developing NEC following CHD surgery had yet to start postoperative feeds [24]. Though feeds are typically initiated within 3 days of surgery [94], Schwalbe–Terilli et al. demonstrated that postoperative CHD infants often suffer from suboptimal caloric intake [107] due to catabolic stresses related to critical illness, increased cardiac load due to shunts, increased pulmonary pressures, and altered gastric absorption [108,109,110]. Additionally, injury to the recurrent laryngeal or vagus nerves, known complications of CHD surgery, can negatively impact feeding [111]. Feeding intolerance in CHD infants is characterized similarly to that of preterm infants, with abdominal distension, increased gastric residuals, and guaiac-positive or bloody stools [99]. Interestingly, preemptive placement of a gastrostomy tube in infants between first and second stage Norwood palliation, in an effort to avoid postsurgical feeding complications, increased survival, but did not improve weight gain or shorten hospital LOS [112]. Prospective studies are needed to generate rigorous feeding recommendations for these high-risk infants.

## 6. Potential Biomarkers for Cardiac NEC

The identification of biomarkers capable of predicting the development of cardiac NEC pre- or postoperatively is, thus far, lacking (Table 2). The use of intestinal fatty acid binding protein (IFABP) as a biomarker for the severity of intestinal ischemic injury secondary to CHD or cardiac surgery has been discussed. Normal turnover of small intestinal enterocytes releases low levels of IFABP into the plasma, but much higher plasma levels of IFABP suggest significant disruption of the small intestinal villi [39]. While also proposed as a biomarker for classical NEC [113,114], circulating IFABP measured 6 h postoperatively has successfully predicted cardiac NEC development in infants undergoing CPB [115]. However, in a prospective study, Pathan et al. found that elevated IFABP, either pre- or postoperatively, poorly predicted clinical outcomes in pediatric CHD cases [39]. Fecal calprotectin, a marker for neutrophil activation, represents another potential biomarker for cardiac NEC. Currently used in the diagnosis of classical NEC [116], fecal calprotectin was significantly increased in DD CHD infants developing NEC postoperatively [117]. Endotoxin activity, an antibody-based assay, has been postulated as a biomarker of intestinal epithelial damage in CHD infants, correlating with levels of C-reactive protein (CRP), lactate level, PICU LOS, and the requirement for inotropes [39], though the risk of NEC development was not specifically addressed in this study. Finally, elevated serum lactate as a proxy for poor splanchnic perfusion has also been suggested as a biomarker of gastrointestinal complications in CHD infants, but a cut-off threshold for this purpose has not yet been established [100]. Given mesenteric hypoperfusion can occur at any time pre- or postoperatively in infants with CHD, identifying biomarkers able to inform on optimal timing of enteral feeds in relation to current gut health is essential.

## 7. Conclusions

Neonates with CHD are at increased risk of intestinal injury due to a combination of mesenteric hypoperfusion, hypoxia-induced inflammation, and surgical stressors. The severity of CHD-associated ischemic injury can vary from ileus to enteritis to cardiac NEC. Low splanchnic blood flow induces endothelial inflammation, an increase in vascular permeability, and immune cell infiltration. Submucosal inflammation spreads to the intestinal epithelium, increasing permeability to noxious antigens and pathogens. The expression of HIF-1α and VEGF, potentially altered by therapeutic oxygen supplementation or abnormal intestinal microvasculature, fails to subdue the excessive inflammatory response. This postulated sequence of mechanistic events, if validated, could aid in differentiating cardiac from classical NEC, and provide novel therapeutic targets. Further studies are also required on best practices encompassing medical and surgical management of this infant population. Recommendations on optimal Stage I HLHS surgical technique or enteral feeding regimens are lacking due to a paucity of multicenter, randomized trials. Identifying diagnostic biomarkers quantitatively scaling to intestinal injury severity is crucial to inform upon best, real-time feeding practices. The high morbidity and mortality associated with cardiac NEC warrant further investigation in an effort to reduce the incidence and severity of this devastating disease.

## Figures and Tables

**Table 1:** Risk factors for cardiac NEC development.

Cardiac NEC Risk Factors
Premature birth (<37 wk gestation)
Low birth weight (<2500 g)
High preoperative risk assessment scores (e.g., RACHS-1)
Red blood cell transfusions
Trisomy 21 diagnosis
Specific CHD pathologies (HLHS, AVSD, TA, APW)

NEC: necrotizing enterocolitis; RACHS-1: risk adjustment for congenital heart surgery; CHD: congenital heart disease; HLHS: hypoplastic left heart syndrome; AVSD: atrioventricular septal defect; TA: truncus arteriosus; APW: aortopulmonary window.

**Table 2:** Potential biomarkers for cardiac NEC.

Potential Cardiac NEC Biomarkers
Intestinal fatty acid binding protein (IFABP)
Fecal calprotectin
Endotoxin activity
Serum lactate

NEC: necrotizing enterocolitis.

## Data Availability

Not applicable.
